# Mitochondrial DNA copy number is associated with psychosis severity and anti-psychotic treatment

**DOI:** 10.1038/s41598-018-31122-0

**Published:** 2018-08-24

**Authors:** Parvin Kumar, Paschalis Efstathopoulos, Vincent Millischer, Eric Olsson, Ya Bin Wei, Oliver Brüstle, Martin Schalling, J. Carlos Villaescusa, Urban Ösby, Catharina Lavebratt

**Affiliations:** 1 Department of Molecular Medicine and Surgery, Karolinska Institutet, Stockholm, Sweden; 2 Center for Molecular Medicine, Karolinska University Hospital, Stockholm, Sweden; 3 Department of Adult Psychiatry, PRIMA Child and Adult Psychiatry AB, Stockholm, Sweden; 4 Department of Neurobiology, Care Sciences and Society, Karolinska Institutet, Stockholm, Sweden; 5 Department of Psychiatry, University of California San Diego, California, USA; 6 Institute of Reconstructive Neurobiology, University of Bonn Medical Faculty, Bonn, Germany

**Keywords:** Prognostic markers, Psychosis, Molecular medicine

## Abstract

Mitochondrial pathology has been implicated in the pathogenesis of psychotic disorders. A few studies have proposed reduced leukocyte mitochondrial DNA (mtDNA) copy number in schizophrenia and bipolar disorder type I, compared to healthy controls. However, it is unknown if mtDNA copy number alteration is driven by psychosis, comorbidity or treatment. Whole blood mtDNA copy number was determined in 594 psychosis patients and corrected for platelet to leukocyte count ratio (mtDNAcn_res_). The dependence of mtDNAcn_res_ on clinical profile, metabolic comorbidity and antipsychotic drug exposure was assessed. mtDNAcn_res_ was reduced with age (β = −0.210, p < 0.001), use of clozapine (β = −0.110,p = 0.012) and risperidone (β = −0.109,p = 0.014), dependent on prescribed dosage (p = 0.006 and p = 0.026, respectively), and the proportion of life on treatment (p = 0.006). Clozapine (p = 0.0005) and risperidone (p = 0.0126) had a reducing effect on the mtDNA copy number also in stem cell-derived human neurons *in vitro* at therapeutic plasma levels. For patients not on these drugs, psychosis severity had an effect (β = −0.129, p = 0.017), similar to age (β = −0.159, p = 0.003) and LDL (β = −0.119, p = 0.029) on whole blood mtDNAcn_res_. Further research is required to determine if mtDNAcn_res_ reflects any psychosis-intrinsic mitochondrial changes.

## Introduction

Psychosis is a common trait in schizophrenia (SZ), schizoaffective disorder, delusional disorder and bipolar disorder (BD)^1^, displayed as a range of symptoms reflecting dissociation from reality. Psychosis and the underlying psychiatric diagnoses have shown a robust association with somatic conditions linked to aging such as cardiovascular disease (CVD)^2^, inflammation^3^, and obesity^4^. These somatic conditions may be caused by chronic oxidative stress^5,6^ or exposure to drug toxicity^7^, leading to cellular ageing^8,9^ which recently has been found increased in psychotic disorders^10,11^.

The mitochondrion is the organelle for ATP production which contains multiple copies of mitochondrial DNA (mtDNA), in blood present in leukocytes and platelets. Ongoing oxidative chain reactions within its compartments make mitochondria a significant source of intracellular reactive oxygen and nitrogen species (ROS/RNS)^12^. The molecular machinery and DNA of the mitochondria are thus at risk from the deleterious effects of oxidative stress resulting in mitochondrial dysfunction^13^.

Mitochondrial dysfunction and ROS/RNS are implicated in the induction of apoptosis through caspase activation^14,15^, inflammasome recruitment^16^ and the activation of downstream cytokines and inflammatory mediators^17^. In the central nervous system, mitochondrial dysfunction could through these processes lead to neurodegeneration, the precursor to cognitive decline and dementia. Mitochondrial dysfunction has been implicated in various somatic and neuro-degenerative disorders^18,19^. Additionally, evidence of mitochondrial dysfunction in BD and SZ has been reported in magnetic resonance spectroscopy studies of small patient groups of BD and SZ^20,21^. Mitochondrial structural abnormalities have been reported in patients with BD^22^ and SZ^23,24^ and both diseases are associated with mtDNA mutations and polymorphisms^25–34^. Hyper-oxidative states and chronic inflammation resulting from mitochondrial dysfunction may be involved in the progression of SZ^6^. Alterations in mtDNA copy number have been studied as a sensitive index of cellular oxidative stress, mitochondrial dysfunction, the aging process^35–38^, and have been associated with a range of factors such as age, gender, smoking and alcohol intake^39–42^. In addition to this, a few studies have reported alterations in leukocyte or whole blood mtDNA copy number in patients of psychotic disorders^10,11,42–44^. Li *et al*., reported lower whole blood mtDNA copy number in antipsychotic drug treatment naïve SZ patients compared to healthy controls, and lower mtDNA copy number in patients being linked to better treatment response^10^. Chang *et al*. showed that leukocyte mtDNA copy number reduction and increased oxidative damage was present in euthymic patients with BD type I (BD-I) compared to healthy controls and De Sousa *et al*. showed that specifically BD-I patients had reduced leukocyte mtDNA copy number compared to BD type II (BD-II) patients and healthy controls^43,44^. Conversely, Cai *et al*. and Tyrka *et al*. showed that childhood adversity as well as depression and anxiety disorders were associated with higher saliva or whole blood mtDNA copy number^11,45^.

Psychosis is common in both BD-I and SZ^46,47^. There are several lines of evidence, for shared disease etiology in SZ and BD, from pharmacology, pathology, family segregation and genetic studies^48–53^. Interestingly, amongst the shared endophenotypes of BD-I and SZ are dysregulation of energy metabolism and mitochondrial dysfunction^20,54,55^. Conversely, patients of bona-fide mitochondrial disorders experience psychotic symptoms and have been misdiagnosed as BD or SZ. This provides more explicit support for the link between mitochondrial dysfunction and psychosis-like symptoms^56–59^.

Anti-psychotic drugs^60^, especially clozapine, are known to increase the risk for obesity and metabolic syndrome (MetS)^7,61,62^. MetS consists of a group of factors, including abdominal obesity, hyperglycemia, dyslipidemia, and hypertension that lead to diabetes and CVD, overrepresented in schizophrenia spectrum of disorders (SSD)^4^ and BD^63^. An *in vitro* study suggested that clozapine causes MetS-like effects at the cellular level by inducing mitochondrial alterations, inflammation and insulin resistance in insulin responsive cells^64^. Intriguingly, while mtDNA copy number reduction have been attributed to MetS^65^, mtDNA alterations caused by antipsychotic drug treatment of patients has to our knowledge not been investigated much in clinical cohorts even if some antipsychotic drugs are known to impair mitochondrial function in various transformed cell lines *in vitro* and in rodent brain *in vivo*^64^. Mitochondria exist in both platelets and leukocytes, and may vary in count and size between subpopulations of leukocytes^66–68^. Platelets skew the mtDNA copy number as on average they have 1.6 molecules of mtDNA with no accompanying nuclear DNA, whereas leukocytes have between 5–30 copies of mtDNA and a nuclear genome^66–68^. Recent specifications for the refinement of mtDNA copy number assessment in whole blood, through correction for leukocyte and thrombocyte count^67,68^, are yet to be adopted by investigators of mtDNAcn in psychosis cohorts^68–70^.

Here, we investigated whole blood mtDNA copy number when corrected for platelet and leukocyte counts, in relation to clinical characteristics in a cohort of psychosis patients, the vast majority of whom were diagnosed with SSD or BD type I. Additionally, the effects of antipsychotic drugs on mtDNA copy number were investigated by using human neurons *in vitro*. In the present study we aimed to determine if mtDNA copy number was associated to (i) severity of psychotic illness, (ii) exposure to specific antipsychotic drugs, and (iii) degree of metabolic comorbidity in psychosis patients. Based on the aforementioned reports, we hypothesized that the mtDNA copy number would be (i) decreased in those with more severe psychosis, (ii) reduced in those taking antipsychotics with reported mitochondrial toxicity, and (iii) reduced with metabolic comorbidity.

## Materials and Methods

### Psychosis patient group

Patients receiving routine clinical treatment from several specialist psychosis clinics were recruited to the Swedish Study of Metabolic Risks in Psychosis (SMRP)^71,72^. The diagnostic and statistical manual of mental disorders, 4th Ed. (DSM-IV) was used to make clinical diagnoses of psychiatric disorders^1^. The evaluation of the severity of mental illness was reported by the clinician according to the clinical global impression-severity scale (CGI-S)^73^, duration of illness, type of current treatment and duration of all treatment received. Medical records were used for the confirmation of medication and dosage. Most patients were treated with anti-psychotics from the onset of syndrome (Supplementary Fig. S1A and B). A self-reported questionnaire in the clinic, under the supervision of a nurse, was used to evaluate somatic health, smoking status and alcohol consumption. Waist circumference and body mass index were measured. The presence of psychiatric disorders in first degree relatives was reported. After an overnight fast, venous blood was collected. Whole blood and serum samples were saved at −80 °C until laboratory analysis. Consecutively recruited patients, n = 614, diagnosed with psychosis were included for the present investigation. The patient characteristics analyzed in this study are presented in Table 1. All participants provided informed consent in writing and ethical approval for the study was received from the Stockholm Regional Ethics Review Board. All methods were performed conforming to applicable guidelines and regulations designated by Karolinska Institutet and the Stockholm Regional Ethics Review Board.

**Table 1:** Demographic and clinical characteristics of the patients with successful genotyping (n = 594).

Patient characteristics	Median (IQR)	Range
Age [years]	44.1 (36.7, 51.2)	18.5–59.3
Body Mass Index (BMI) [kg/m^2^]	27.9 (24.5, 31.8)	11.2–51.32
Length of hospital stay [months]	4 (3, 4)	1–7
Alcohol consumption [g/week]	16 (0, 89)	0.0–216
Age at onset [year]	26 (21, 32)	9.0–56.0
Clinicians Global Impression (CGI)- Severity	4 (3, 4)	1–7
Duration of antipsychotic treatment [years]	13.0 (6.0, 22.0)	0.0–43.0
Duration of illness [years]	14.8 (8.0, 23.3)	0.2–43.6
	**n**	**%**
Gender		
Male	336	56.7
Female	258	43.4
Psychosis onset		
Early psychosis onset (≤18 yr old)	62	10.4
Late onset (>18 yr old)	452	76.9
Unknown	75	12.6
Smoking		
Yes (every day or sometimes)	239	40.2
No (never or quit)*	298	50.1
Unknown	57	9.6
Presence of psychiatric disease in first-degree relative		
Yes	201	33.8
No	318	53.5
Unknown	75	12.6
Main psychiatric diagnoses		
Schizophrenia	306	51.5
Schizoaffective disorder	49	8.2
Delusion	26	4.4
Psychosis unspecified	84	14.1
Bipolar disorder	26	4.4
Other	49	8.2
Undiagnosed psychosis	54	17.8
Antipsychotic medication		
None	60	10.1
Multitherapy	42	7.1
Risperidone	91	15.3
Olanzapine	108	18.2
Zuclopenthixol	48	8.1
Perphenazine	56	9.4
Haloperidol	49	8.2
Clozapine	57	9.6
Aripiprazole	73	12.3
Quetiapine	33	5.6
Ziprasidone	17	2.9
Flupentixol	16	2.7
Unknown	83	14.0
Mood stabilizers	65	10.9

Clinical Global Impression–Severity (CGI-S) was available from 513 of the patients. *Of this group, ~45% had quit smoking.

Whole blood mtDNA copy number, adjusted for platelet to leukocyte count ratio, of the patients were investigated for association with gender, age, CGI-S score, smoker status, alcohol consumption, and psychiatric diagnosis together with antipsychotic drug treatment, presence of mood stabilizer, and with variables associated with the metabolic profile of patient including waist circumference, presence of high blood pressure, fasting blood levels of high-density-lipoprotein (HDL), low-density-lipoprotein (LDL), insulin sensitivity marker (HOMA-IR): homeostatic model of assessment for insulin resistance^74^, glucose levels, and presence of metabolic syndrome (MetS). Gender, smoking status, presence of antipsychotic and mood stabilizing drugs, presence of high blood pressure and MetS were dichotomous variables while all others were continuous variables. Non-smokers were designated as those who have not smoked at all and all others were considered smokers. MetS and high blood pressure were defined according to International Diabetes Federation (IDF) criteria(61).

### Measurement inflammation and metabolic markers in the psychosis patients

Serum lipid profiles (HDL and LDL), plasma glucose, insulin levels and blood cell counts, were determined according to standard GLP protocols. Analyses were performed at the clinical laboratory of the Karolinska University Hospital.

### Whole blood DNA extraction and mitochondrial DNA copy number (mtDNA)

DNA was extracted from venous blood using a standard phenol-chloroform method (Lindblom and Holmlund)^75^ followed by desalting using Illustra NAP-5 columns (GE Healthcare, Buckinghamshire, UK) and was quantified spectrophotometrically with the NanoDrop ND-1000 spectrophotometer (NanoDrop Technologies, Wilmington, DE, USA).

The copy number of mitochondrial DNA per nuclear genome (i.e. per cell) was determined using real-time quantitative PCR (qPCR) according to Rooney JP^76^ and Venegas V^77^ protocol. The relative amount of mitochondrial gene *tRNA-Leu*(UUR), to the nuclear single-copy gene β2-microglobulin (*B2M)* (mtDNA copy number) was determined using a standard curve. In brief, each DNA sample (4.0 ng) was assessed for *tRNA-Leu*(UUR) and *B2M* in triplicate within the same 384-well plate, amplified by using Power SYBR Green in 10 µl total reaction volume. The reaction was performed on QuantStudio 7 Flex (Applied Biosystems; Life Technologies; Thermo Fisher Scientific, Waltham, MA, USA) with the following conditions: 95 °C for 15 min, followed by 40 repeats of 95 °C for 15 s and 60 °C for 1 min, followed by a dissociation stage to monitor amplification specificity. The same standard curve of control genomic human DNA (Applied Biosystems) ranging from 10 ng to 0.016 ng, was run on each plate for both genes and was used to determine the quantity of each gene for each sample. This allowed controlling for differences in the efficiencies between that of mtDNA and *B2M*. The gene quantities were then used to determine the M/S ratio for each sample. DNA samples with a Ct standard deviation of ≥0.3 between triplicates or a Ct value outside the standard curve were omitted from the analyses. Samples were analyzed in 12 consecutive plates. R^2^ coefficients of the standard curves were above 0.99 for each primer set and 384-plate. The inter-plate coefficient of variation (CV) of M/S ratio was 8.3% calculated as a mean from three inter-plate control samples run in the 12, 384-well plates. The mean intra-plate CV of M/S ratio of the three control samples in triplicates was 4.6%. Primer binding regions were selected for low deletional <3%^78^ or mutational (SNPs) exposure 2.2%^79^. No known psychiatry related SNPs are present in the primer binding mtDNA regions (Online Mendelian Inheritance in Man, OMIM®^80^ and www.mitomap.org)^79^. The probability of random somatic mutations to occur at the primer binding regions is calculated to be 0.24%. For a subset of the patients an alternative amplicon in the D-loop region of the mitochondrial genome was targeted for the quantification of mtDNA to confirm the sensitivity of the assay. This D-loop amplicon was selected based on Bai and Wong^78^ and was carefully designed from mtDNA sequences that do not contain reported mutations or polymorphisms occurring in more than 1.6% of the population (http://www.genpat.uu.se/mtDB/Polysites)^79,81^. The D-loop region has been used for mtDNA content analysis in similar investigations for mtDNA copy number quantification^11,42^. mtDNAcn measurements targeting the D-loop and *tRNA-Leu*(UUR) were compared and found to be highly correlated, n = 55, r = 0.996, p = 1.62E-58.

The primer sequences were (written 5′ → 3′): mtDNA [*tRNA-Leu*(UUR)] Fw: CAC CCA AGA ACA GGG TTT GT; mtDNA Rv: TGG CCA TGG GTA TGT TGT TA; *B2M* Fw: TGC TGT CTC CAT GTT TGA TGT ATC T; *B2M* Rv: TCT CTG CTC CCC ACC TCT AAG T. Alternative primer binding region in D-loop of mitochondrial genome (written 5′ → 3′): D-loop Fw: CAT CTG GTT CCT ACT TCA GGG; D-loop Rv:TGA GTG GTT AAT AGG GTG ATA GA.

The mtDNA copy number measurement success rate was 97% (594/614 samples). mtDNA copy number was corrected for platelet and leukocyte count as these variables are known to affect MS ratio. Platelets have mitochondria and accompanying mtDNA but no nuclei and hence lack a nuclear genome, which results in an overestimation of mtDNA^68^. Whole blood derived mtDNA are associated with leukocyte count as reported by several groups and thus a correction for the platelet and leukocyte have been suggested as a refinement to the mtDNA copy number measurement^68–70^.

### Generation of human neurons *in vitro* and drug treatment

The long-term neuroepithelial stem cell (NESC) line I3.2, derived from human embryonic stem cells and previously described^82^ was used for the generation of human neurons *in vitro*. For maintenance, NESC were cultured on poly-l-Ornithine (PLO, Sigma-Aldrich, Irvine, UK) and laminin (Sigma-Aldrich, Irvine,UK) coated wells, in DMEM/F12 (Gibco) media supplemented with N2 and B27 supplements (Life Technologies; Carlsbad, CA, USA) in the presence of the growth factors EGF and FGF2-basic as previously described^82^. To induce differentiation, EGF and FGF2-basic were removed from the media (Supplementary Fig. 2A and B). Briefly, NESC were seeded on PLO/laminin at the density of around 75000–150000 cells per well, in a 48-well plate, and cultured for 7 days in DMEM/F12 media supplemented with N2 (1:100) and B27 (1:100) supplements. After one week of differentiation, media was changed to DMEM/F12 supplemented with N2 (1:100) with the addition of glial cell-derived neurotrophic factor (GDNF) 20 ng/ml, brain-derived neurotrophic factor (BDNF) 20 ng/ml, ascorbic acid 10 mM, dibutyryl adenosine 3′,5′-cyclic monophosphate sodium salt (dcAMP) 25 mM. Drug treatments started after 2 weeks of *in vitro* differentiation, when NESCs had a clear neuronal morphology. Differentiated neurons were characterized through immunohistochemistry stains for neuronal marker TUBB3.

For drug treatments, neurons were treated in plain DMEM/F12 media supplemented with only N2 (1:100), with the addition of either the vehicle or different concentrations of the drugs for one week before they were processed for DNA extraction. Clozapine and risperidone were dissolved in 1 M HCl before added to cell culture medium in the concentrations of 0.075 μM, 0.75 μM and 0.025 μM, 0.25 μM respectively. The vehicle used was plain DMEM/F12 media acidified by 0.0025 μM HCl, pH neutralized by CO_2_ buffer. The concentrations were selected to simulate clinical target concentrations in plasma and diluted by 10-fold to simulate concentration in the cerebrospinal fluid and brain interstitial fluid^83–85^. At the end of treatment, cells were lysed and DNA extraction was performed using the Quick-DNA™ Miniprep Plus Kit (Zymo Research Corp, Irvine, CA, USA). Subsequently mtDNA copy number determination was performed as described above. Experiments were run in 3 biological replicates.

### Statistical analyses

The effect of platelet and leukocyte counts on mtDNA copy number (mtDNAcn) was assessed by regressing mtDNAcn on age, platelet count and leukocyte count as follows: mtDNAcn = b_0_ + b_1_ (age) + b_2_ (leukocyte count) + b_3_ (platelet count). Thereafter, mtDNAcn was corrected for platelet and leukocyte counts by regressing mtDNAcn on the platelet to leukocyte count ratio (platelet count/leukocyte count), as outlined in Hurtado-Roca, *et al*.^68^, generating normally distributed unstandardized residuals. The unstandardized residual variable, hereafter designated as mtDNAcn_res_, was treated as the dependent variable in the following regression analyses.

We evaluated the significance of psychiatric diagnosis on mtDNAcn_res_ using an analysis of covariance (ANCOVA) adjusting for covariates suggested to influence mtDNAcn_res_, i.e. age, gender, smoking, alcohol intake and psychosis severity^10,39–42,86,87^. Non-parametric Mann Whitney U test, was used to assess difference in mtDNAcn_res_ between patients with MetS and those without MetS.

To study putative predictors of mtDNAcn_res_, regression modelling was performed as previously described^72,88^. In brief, an iterative method of regression modelling was initiated with no variables in the model, adding each variable to be tested with an entry requirement of p < 0.05 and a loss of significance at p > 0.10. Regression residuals were ensured to be normally distributed. Errors due to multiple testing were corrected for using the bonferroni method. Four multiple linear regression models were built to evaluate the effects of i) age, gender, psychosis severity (CGI-S), alcohol intake and smoking ii) drug treatment and iii) metabolic factors on mtDNAcn_res_. Model 1 was as follows: mtDNAcn_res_ = b_0_ + b_1_ (age) + b_2_ (gender) + b_3_ (CGI) + b_4_ (smoking) + b_5_ (alcohol) + b_6_ (psychiatric diagnosis). The second model looking at the effect of antipsychotic drugs and mood stabilizers on mtDNAcn_res_ was as follows: mtDNAcn_res_ = b_0_ + b_1_ (age) + b2 (gender) + b_3_ (psychiatric diagnosis) + b_4_ (mood stabilizer) + b_n_ (drug), where (drug) represents the patient’s treatment [yes/no] with any of n = 11 different antipsychotics. The mood stabilizer variable represents the presence of any mood stabilizer [yes/no] as more detailed information was not available. The numbers of patients on each drug and on any mood stabilizer are listed in Table 1. The mtDNAcn_res_ of patients using a drug significant in model 2 were, for confirmation, compared to that of the all other patients using Mann-Whitney U test. The effect of clozapine and risperidone treatment on mtDNAcn_res_ was further investigated by calculating Spearman’s correlation coefficient, ρ, between the prescribed daily drug dose and the mtDNAcn_res_. To compare the effects of CGI-S with antipsychotic drug treatment effects we imported the significant variables from the model 1 and 2 to a third regression model. The equation of model 3 was as follows: mtDNAcn_res_ = b_0_ + b1 (proportion of life on antipsychotic treatment) + b2 (CGI) + b3 (age). This model was separately run on patients who were treated with clozapine or risperidone (model 3a, n = 139) and patients who were not on risperidone and clozapine (model 3b, n = 348). The fourth model (metabolic profile) which analyzed the effect of metabolic factors was adjusted for significant predictors of mtDNAcn_res_ obtained from model 3. Model 4a was mtDNAcn_res_ = b_0_ + b_1_ (age) + b_2_ (proportion of life on antipsychotic treatment) + b_3_ (gender) + b_4_ (waist) + b_5_ (LDL) + b_6_ (HDL) + b_7_ (glucose) + b_8_ (Log_10_HOMA-IR) + b_9_ (high BP); and model 4b was mtDNAcn_res_ = b_0_ + b_1_ (age) + b_2_ (CGI) + b_3_ (gender) + b_4_ (waist) + b_5_ (LDL) + b_6_ (HDL) + b_7_ (glucose) + b_8_ (Log_10_HOMA-IR) + b_9_ (high BP). Model 4a was run on patients who were treated with clozapine and risperidone (n = 123) and model 4b was run on patients who were not on risperidone and clozapine (n = 324). A sensitivity analysis of the Models 1, 2, 3 and 4 was performed in the patients with a diagnosis of schizophrenia. Output model indices and lack of multi-collinearity between input variables were confirmed using standard multiple linear regression (the enter method), including all input variables. Analyses were performed using IBM Statistical Package for the Social Sciences version 23, (IBM Corporation, USA). Power calculations were performed using http://biomath.info/power/ttest.htm.

## Results

As previously reported we found that whole blood mtDNAcn was significantly influenced by platelet and leukocyte counts (β_platelets_ = 0.119, p = 0.005, and β_leukocytes_ = −0.298, p < 0.001). A regression of whole blood mtDNAcn on the platelet to leukocyte count ratio (platelet/leukocyte) was performed to generate normally distributed unstandardized residuals (mtDNAcn_res_) which were used in the following analyses as a dependent variable (Supplementary Fig. S3).

The clinical characteristics of the psychosis patients are shown in Tables 1 and 2. The psychiatric diagnoses within the psychosis patient cohort were SZ, schizoaffective disorder, delusional disorder, psychosis unspecified, BD and other disorders with psychotic features. The effect of diagnosis on mtDNAcn_res_ was evaluated using an analysis of covariance (ANCOVA) correcting for covariates suggested to influence mtDNAcn_res_, i.e. age, gender, smoking and alcohol intake. No significant difference in mtDNAcn_res_ was observed between the six diagnosis groups (p = 0.212). Nonetheless, in subsequent regression analyses (Models 1, 2, 3 and 4) of the psychosis patients, adjustment was made for psychiatric diagnosis, in addition to age and gender, to exclude detectable confounding. Moreover, a sensitivity analysis in only those with a SZ diagnosis (n = 306) replicated the findings in Models 1–4 of the full psychosis cohort (Supplementary Table S1).

**Table 2:** Metabolic characteristics of the psychosis patients with successful genotyping (n = 594).

Metabolic characteristics	Median (IQR)	Range	Reference
Men (MetS 42.0%, high BP 63.0%)			
Waist circumference [cm]	104 (93–113)	69–150	<94
HDL-cholesterol [mmol L^−1^]	1.0 (0.9–1.2)	0.200–3.50	>1.03
LDL-cholesterol [mmol L^−1^]	3.5 (2.9–4.0)	0.0–5.8	<2.6
Fasting plasma glucose [mmol L^−1^]	5.3 (4.9–5.9)	3.20–18.7	<5.6
Log HOMA-IR	0.64 (0.49–0.90)	0.098–1.67	<2.00
Women (MetS 40.2%, high BP 50.2%)			
Waist circumference [cm]	95 (85–104)	65–135	<80.0
HDL-cholesterol [mmol L^−1^]	1.3 (1.1–1.6)	0.70–2.80	>1.29
LDL-cholesterol [mmol L^−1^]	3.5 (2.9–4.2)	1.4–7.0	<2.6
Fasting plasma glucose [mmol L^−1^]	5.3 (4.8–5.8)	3.8–23.6	<5.6
Log HOMA-IR	0.60 (0.41–0.79)	0.059–1.76	<2.00

MetS: Metabolic syndrome according to International Diabetes Federation (IDF) criteria(61).

High BP: Elevated blood pressure according to IDF criteria(61).

IQR: interquartile range.

HDL: high-density-lipoprotein.

LDL: low-density-lipoprotein.

Log HOMA-IR: log10 of homeostatic model of assessment for insulin resistance.

### Clinical Profile (Model 1): mtDNA copy number was associated with psychosis severity (CGI-S)

**Figure 1:**
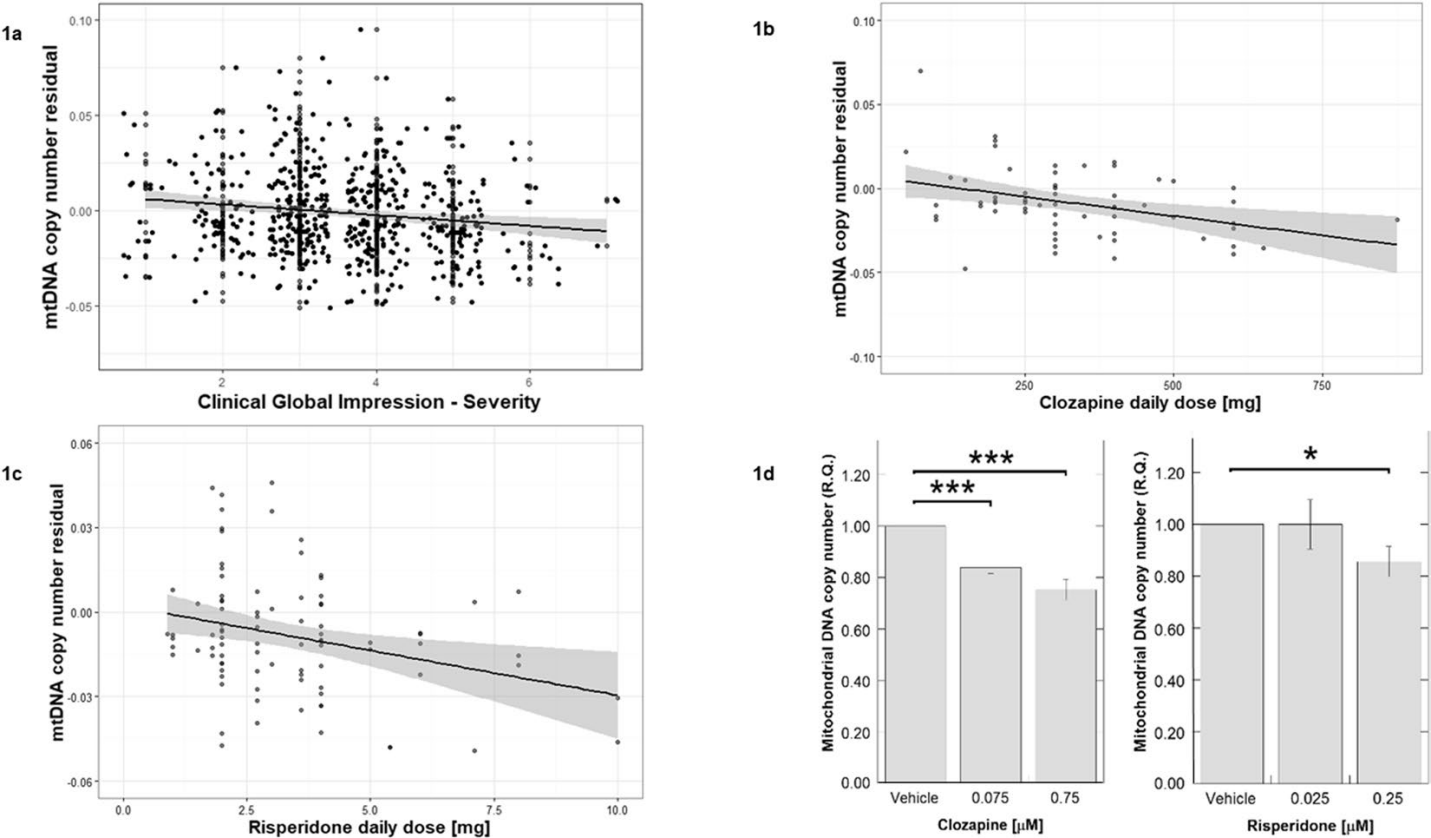
(**a**) mtDNAcn_res_ was negatively correlated with Clinical Global Impression - Severity (CGI-S) score (Spearman’s ρ = −0.152, p = 0.0006, n = 507). (**b**) mtDNAcn_res_ correlated with the prescribed daily dose of clozapine (Spearman’s ρ = −0.351, p = 0.006, n = 61). (**c**) mtDNAcn_res_ correlated with the prescribed daily dose of risperidone (Spearman’s ρ = −0.233 p = 0.026, n = 91). (**d**) Clozapine treatment, at 0.075 μM there was a 16% reduction in mtDNA copy number compared to vehicle treated cells (p = 0.0005), and at 0.75 μM there was a 25% reduction in mtDNA compared to vehicle treated cells (p = 0.0004). Risperidone treatment, at 0.025 μM there was no change in mtDNA copy number compared to vehicle treated cells, whereas at 0.25 μM there was a 14% reduction in mtDNA copy number compared to vehicle treated cells (p = 0.0126). Error bars indicate standard error. R.Q.: relative quantification (mtDNA copy number). *p < 0.05; ***p < 0.001.

Association between mtDNAcn_res_ and severity of psychotic illness, the latter indicated by the clinician-rated CGI-S score, was assessed. A multiple linear regression model including the predictors: age, gender, CGI, smoking, alcohol, psychiatric diagnosis, returned the variables age and CGI as significant predictors of mtDNAcn_res_ (adjusted R^2^ = 0.038, F = 10.7, p < 0.001, n = 497). Increasing age and psychosis severity (CGI-S) were associated with a decreased mtDNAcn_res_ (age: β_standardized_ = −0.142, t = −3.20, p < 0.001; CGI: β_standardized_ = −0.128, t = −2.90, p = 0.004) (Table 3). A linear relationship between mtDNAcn_res_ and CGI-S was observed (Spearman’s correlation coefficient ρ = −0.152, p = 0.0006, n = 507, Fig. 1a), and between mtDNAcn_res_ and age (Spearman’s ρ = −0.188, p < 0.001).

**Table 3:** Final models of clinical parameters associated with whole blood mitochondrial DNA copy number adjusted for platelet to leukocyte count.

Variable	B	SE	β_standardized_	t	p
Model 1: Clinical profile (adjusted R^2^ = 0.038, ANOVA: F = 10.7, n = 497)					
(Constant)	0.029	0.007		4.51	<0.001
Age	<0.001	<0.001	−0.142	−3.20	0.001
CGI	−0.003	0.001	−0.128	−2.90	0.004
Model 2: Treatment (adjusted R^2^ = 0.064, ANOVA: F = 9.61, n = 504)					
(Constant)	0.03	0.006		4.91	<0.001
Age	−0.001	<0.001	−0.210	−4.74	<0.001
Clozapine	−0.010	0.004	−0.110	−2.52	0.012
Risperidone	−0.009	0.003	−0.109	−2.48	0.014
Model 3 Group A: Treatment vs psychosis severity (adjusted R^2^ = 0.047, ANOVA: F = 7.70, n = 136)					
(Constant)	0.002	0.004		0.402	0.689
Proportion of life on treatment	<0.001	<0.001	−0.232	−2.78	0.006
Model 3 Group B: Treatment vs psychosis severity (adjusted R^2^ = 0.042, ANOVA: F = 8.46, n = 340)					
(Constant)	0.039	0.009		4.39	<0.001
Age	−0.001	<0.001	−0.159	−2.97	0.003
CGI	−0.004	0.001	−0.129	−2.41	0.017
Model 4 Group A: Metabolic profile (adjusted R^2^ = 0.039, ANOVA: F = 5.93, n = 123)					
Proportion of life on treatment	<0.001	<0.001	−0.215	−2.44	0.016
Model 4 Group B: Metabolic profile (adjusted R^2^ = 0.055, ANOVA: F = 7.13, n = 319)					
(Constant)	0.043	0.009		4.56	<0.001
Age	−0.001	0.001	−0.173	−3.17	0.002
CGI	−0.003	0.002	−0.113	−2.07	0.040
LDL	−0.001	<0.001	−0.119	−2.19	0.029

CGI: Clinical Global Impression-Severity.

Group A: patients receiving clozapine or risperidone.

Group B: patients not receiving clozapine or risperidone.

### Treatments (Model 2): Clozapine and risperidone treatments were associated with decreased mtDNA copy number

The effect of antipsychotics on mtDNAcn_res_ was assessed using multiple linear regression modelling including the variables age, gender, psychiatric diagnosis, presence of any mood stabilizer [yes/no] and the different antipsychotic drugs [yes/no]. Age, clozapine and risperidone were significant predictors of mtDNAcn_res_ (adjusted R^2^ = 0.064, F = 9.61, p < 0.001, n = 504). Age, clozapine and risperidone were associated with decreased mtDNAcn_res_ (age: β_standardized_ = −0.210, t = −4.74, p < 0.001, clozapine: β_standardized_ = −0.110, t = −2.52, p = 0.012, risperidone: β_standardized_ = −0.109, t = −2.48, p = 0.014) (Table 3). To confirm the difference in mtDNAcn_res_ for clozapine and risperidone treatment groups compared to patients not on these treatments non-parametric Mann-Whitney U tests were performed. Both the clozapine and risperidone patient groups showed a significantly reduced level of mtDNAcn_res_ (clozapine: p = 0.022 and risperidone: p = 0.005). Furthermore, clozapine and risperidone daily oral treatment doses correlated with mtDNAcn_res_ (clozapine: Spearman’s ρ = −0.351, p = 0.006; risperidone: Spearman’s ρ = −0.233, p = 0.026, Fig. 1b,c). As expected, there was no correlation for any of the other antipsychotic drugs with mtDNAcn_res_ corrected for age (Spearman’s |ρ| < 0.074, p > 0.085).

### Psychosis severity and proportion of life on antipsychotic treatment (Model 3): The effect of clozapine and risperidone treatment duration superseded the effect of psychosis severity on mtDNA copy number

Since there was an association for clozapine and risperidone treatment to mtDNAcn_res_, we assessed the effect of proportion of life on antipsychotic treatment, compared to the effect of psychosis severity (CGI-S) and age detected in model 1. First, proportion of life on treatment correlated with CGI-S to a similar extent for both groups of patients (those on clozapine or risperidone: Spearman’s ρ = 0.258, p = 0.002, n = 140; those patients not on clozapine or risperidone: ρ = 0.234, p < 0.001, n = 349; Supplementary Fig. 4A and B), with relatively small effect sizes in both subgroups (β = 0.248 and 0.251 respectively). Second, we compared the effect on mtDNAcn_res_ by psychosis severity (CGI-S), age and the proportion of life on treatment. For those patients on clozapine and risperidone the model (3a) explained 4.7% of the variance in mtDNAcn_res_ (adjusted R^2^ = 0.047, F = 7.70, p = 0.006, n = 136, with proportion of life on treatment being the single predictor (β_standardized_ = −0.232, t = −2.78, p = 0.006, n = 136). However, for the patients who were not on risperidone or clozapine, the model (3b) explained 4.2% of the variance, age and CGI-S were the only significant predictors: adjusted R^2^ = 0.042, F = 8.46, p < 0.001, n = 340; age: β_standardized_ = −0.159, t = −2.97, p = 0.003 and CGI-S: β_standardized_ = −0.129, t = −2.41, p = 0.017 (Table 3); thus the proportion of life on treatment did not additionally explain variance in mtDNAcn_res_.

### The association of mtDNA with metabolic profile (Model 4)

mtDNAcn_res_ was tested for association with the metabolic profile of patients as described by the variables waist circumference, fasting blood levels of LDL, HDL, glucose, log_10_HOMA-IR and presence of high BP. The multiple linear regression model to identify potential metabolic predictors of mtDNAcn_res_ was corrected for the variables detected to influence mtDNAcn_res_ in models 3a and 3b. Therefore, modelling for those on clozapine or risperidone was adjusted for the proportion of life on treatment (model 4a) whereas the model for the patients not on clozapine or risperidone included age and CGI-S (model 4b). Model 4a (adjusted R^2^ = 0.039, F = 5.93, p = 0.016), returned proportion of life on treatment as a significant predictor of mtDNAcn_res_ variance (β_standardized_ = −0.215, t = −2.44, p = 0.016), where metabolic variables did not explain any variance of mtDNAcn_res_. Model 4b (adjusted R^2^ = 0.055, F = 7.13, p < 0.001), returned the significant variables age (β_standardized_ = −0.173, t = −3.17, p = 0.002) and CGI-S (β_standardized_ = −0.113, t = −2.07, p = 0.040) and LDL (β_standardized_ = −0.119, t = −2.19, p = 0.029) (Table 3). There was no association between presence of MetS and mtDNAcn_res_ (Mann-Whitney U test: p = 0.130).

### Antipsychotic drug effects on mtDNA copy number in human neurons *in vitro*

To assess the effect of clozapine and risperidone on neurons, human neurons generated *in vitro* from NESCs were exposed for 7 days to clozapine (0.075 μM and 0.75 μM) or risperidone (0.025 μM and 0.25 μM). For clozapine treatment, at 0.075 μM, there was a 16% reduction in mtDNA copy number compared to vehicle treated cells (p = 0.0005), and at 0.75 μM, the corresponding reduction was 25% (p = 0.0004) (Fig. 1d). For risperidone treatment, at 0.025 μM there was no change in mtDNA copy number compared to vehicle treated cells, but at 0.25 μM, there was a 14% reduction in mtDNA copy number compared to vehicle treated cells (p = 0.0126) (Fig. 1d). Thus, risperidone was found to have a reducing effect on mtDNA copy number at a concentration simulating the clinical target level in plasma but not at the concentration simulating CSF or brain interstitial target level. Clozapine was found to be associated with reduced mtDNA copy number at both doses, in a dose dependent manner. Higher doses of anti-psychotic drugs (clozapine, 75 μM and risperidone, 25 μM) were associated with massive neuronal cell death (data not shown).

## Discussion

From a neuroanatomical point of view, a much reviewed discrepancy between SZ and healthy controls are aberrations in dendritic spine morphology. These have been observed in the cortical layers of SZ patients with concomitant increases in the molecular signatures of mitochondrial dysfunction^89,90^. Interestingly, compelling evidence exists to explain the link between mitochondrial dysfunction and aberrations in dendritic spine morphology as mitochondria play salient roles in dendritic spine architecture and neuronal processes which affect crucial cortical circuitry^91,92^. Mitochondrial dysfunction and oxidative stress in both brain and leukocytes are overrepresented in not only SZ but also BD type I^23,26,44,93^. There is a link between mitochondrial dysfunction and psychosis-like symptoms^53,94^, and mitochondrial dysfunction is reported to be intrinsic to the complex etiology of SZ^89^. Oxidative stress induced in dysfunctional mitochondria can cause deletions of mtDNA, and influence mitochondrial biogenesis^36^. A few studies have utilized mtDNA copy number analysis to investigate mitochondrial dysfunction and reported reduced whole blood or leukocyte mtDNA copy number in SZ and BD type I compared to healthy controls^10,43,44^.

Similarly, the present study was performed to further explore whole blood mtDNA copy number, adjusted for platelet to leukocyte count ratio, in psychosis by focusing on effects of disease severity, antipsychotic drug treatment and metabolic comorbidity. The main findings of this study were that (i) mtDNA copy number was reduced with increasing psychosis severity, and (ii) the antipsychotic drugs clozapine and risperidone decreased mtDNA copy number in patient blood with similar effects on human neurons *in vitro* in a dose dependent manner. While this has not previously been reported, clozapine, risperidone and other antipsychotic drugs are known to be toxic to mitochondria in various transformed cell lines (neuroblastoma, adipocytes, myoblasts, hepatocytes, lymphoblasts and monocytes) *in vitro*^64,89^ and in rodent brain^95^. Accordingly, we found reduced whole blood mtDNA copy number in psychosis patients treated with clozapine and risperidone compared to psychosis patients treated with or without other antipsychotics. We also showed an inverse correlation between prescribed oral drug dosage and mtDNA copy number. The proportion of life on antipsychotic treatment was a significant predictor of mtDNA copy number variance only in the subset of patients who were treated with clozapine and risperidone.

For patients not on clozapine or risperidone, decreasing mtDNA copy number was associated with increasing age and psychosis severity measured by the CGI-S (n = 348). Existing literature from studies of healthy individuals and non-psychotic post mortem studies support our finding that mtDNA copy number is reduced with advancing age^96–98^. Where psychotic features are of concern, conclusions along a similar grain were made by Li *et al*.^10^, who reported reduced whole blood mtDNA copy number in first-episode drug-naïve SZ patients (n = 137) compared to healthy controls and a trend for reduced mtDNA copy number to be associated with positive symptoms in SZ patients (p = 0.07). Two other studies which considered BD patients found reduced leukocyte mtDNA copy number in euthymic BD-I patients compared to BD-II and healthy controls^43,44^.

The findings linking psychosis with mtDNA copy number are bolstered by previous human post mortem studies where the number of mitochondria per tissue volume was found to be significantly decreased in oligodendrocytes of prefrontal cortex and caudate nucleus of SZ patients^23^. Mitochondrial density in the neuropils were significantly reduced in the caudate nucleus and putamen of SZ subjects compared to controls^93,99^. Moreover, psychotic positive symptoms, such as hallucinations, have been reported in subjects with mitochondrial disease^94^, and a high degree of mitochondrial myopathy encephalopathy lactic acidosis and stroke-like (MELAS) episodes have been reported in SZ and BD^31^.

To explore if the effects of clozapine and risperidone on mtDNA copy number in blood cells could also be detected in human neurons, we treated NESC-derived human neurons with clozapine and risperidone. We found that the effect was dose dependent at drug concentrations corresponding to target CSF levels during therapy^84,100^. Clozapine had a stronger effect than risperidone on mtDNA copy number at drug levels which simulate CSF or brain interstitial target levels. At clinical target plasma concentrations, there was a significant decrease (estimated at 15–25% reduction) in mtDNA copy number in the neurons exposed to any of the two drugs. These findings are supported by several studies that have shown that antipsychotics (clozapine, risperidone, haloperidol, olanzapine, quetiapine, chlorpromazine, and thiothixene) can inhibit the mitochondrial respiratory chain and cause further damage to mitochondria through oxidative stress^95,101,102^, however, the effect on mtDNA copy number was not previously studied. We detected an effect by only clozapine and risperidone but had a statistical power corresponding to 80% to detect an effect similar to that of risperidone for those drugs with a sample size of above 40 (that is haloperidol, olanzapine, zuclopenthixol, aripiprazole, perphenazine, risperidone and ziprasidone). Thus, we did not confirm a previously reported mitochondrial effect for haloperidol and olanzapine. An important limitation of this study is that while the total length of antipsychotic drug treatment was known, the specific antipsychotic drug names were available only for the antipsychotic drug treatment at sampling, and not historically. However, using prospective data starting at sampling we estimate that approximately 10% switched to another antipsychotic drug in a year. If mtDNA copy number depletion and accelerated cellular ageing occur as a result of these antipsychotic treatments, further investigation on mitochondria dysfunction as mediator between antipsychotics and associated comorbidities may be warranted. Another limitation is that the type of mood stabilizers used, in the 11% of patient cohort who were treated with them, was unknown. Amongst mood stabilizers, lithium has been shown to have beneficial effects on mitochondria through enhanced oxidative phosphorylation^103^ and restoring defunct vacuolated mitochondria to their healthy baseline structure^104^. A reduction of mtDNA observed in lithium treated *C*. *elegans* with increased longevity, a finding going against the grain of understanding that a reduction of mtDNA signals cellular ageing, which was possibly explained by increasingly efficient mitochondrial bioenergetics^105^. Valproic acid, similarly has been reported to enhance mitochondrial biogenesis, accompanied with an increase in mtDNA copy number in hepatocytes^106^. Conversely lamotrigine an anti-convulsant and mood stabilizer used in more acute cases was reported to have toxic effects on mitochondria^107^.

Metabolic comorbidity and inflammation are gaining ground as bona-fide hallmarks of psychotic disorders^4^. It has been previously reported that metabolic syndrome per se is associated with a reduced mtDNA copy number^65^. We detected an association for blood mtDNA copy number to level of LDL, similarly strong as that to CGI-S in the psychosis patients not on clozapine or risperidone. The effect of metabolic syndrome on mtDNA copy number and mitochondrial functionality could be enhanced and mediated by systemic inflammation^108^. Therefore, a limitation of the present study is the lack of quantified inflammatory markers.

We adjusted the mtDNAcn for platelet to leukocyte ratio, but not for subpopulations of leukocytes as their counts were unavailable. While we cannot exclude that our observations might be due to changes in relative WBC subpopulation ratios, existing literature suggests that in a large patient group, total WBC count and platelet count capture the relevant information we need to correct for when making conclusions on changes in peripheral mtDNA^69,70,109^. Variation between subpopulations are rarely accounted for in mtDNAcn analyses. The mtDNA copy number variance explained by significant predictors was low, 4–6%. However, this is comparable with previous studies investigating the relationship between psychiatric disorders and cellular ageing markers (depression and telomere length)^110–112^. Several association tests were performed which requires correction for multiple testing. However, the regression model reported (models 1, 2 and 4 (model 3 building on 1 and 2)) were designed to test individual hypotheses. Within each model, the findings we report passed a multiple testing correction according to Bonferroni.

The focus of our study was to investigate the relationship between mtDNA copy number, psychosis severity and antipsychotic drug treatment in a psychosis outpatient clinic setting. Here the appropriate controls would have been anti-psychotic drug treatment naïve psychosis patients. However, in the Swedish healthcare setting patients who are diagnosed with the schizophrenia spectrum of disorders are always offered treatment. Therefore we were not able to obtain material from age and gender matched drug-naïve patients. A further limitation is that our study lacks healthy controls, however, previous comparisons performed have reported mtDNA copy number to be reduced in anti-psychotic treatment naïve patients of psychotic disorders (SZ and BD-I) compared to healthy controls^10,43,44^.

The patient group in our study belongs to multiple DSM-IV-based diagnostic categories, but all patients showed symptoms of severe psychosis and experienced impaired functionality. A unifying criteria of the study cohort was that, all patients were recruited from specialist psychosis outpatient clinics in Sweden, where they received anti-psychotic treatment, unless refused. Our decision to include data from all psychosis patients regardless of diagnoses is in accordance with the Research Domain Criteria (RDoC) paradigm. The RDoC is based on domains of human behavior and functionality e.g cognitive impairment in the presence of psychotic symptoms^113^. The presence of psychotic symptoms are relevant to the cognitive systems domain of the RDoC matrix, regardless of the DSM-IV criteria based diagnosis^114^.

To further address the heterogeneity of the patient cohort from the DSM-IV perspective we performed a sensitivity analysis by repeating linear modelling on the subset of the patients who had a SZ diagnosis. The main findings of the study were well replicated in the SZ cohort. Furthermore, we found no statistically significant effect of the DSM-IV-based diagnoses mtDNA copy number levels, even if this may have been due to a limited sample size within certain diagnostic groups i.e BD or delusion. In place of a symptom severity scale such as the positive and negative symptom scale (PANSS), which was unavailable in this study, we used the clinician-rated CGI-S, an indication of the patients’ mental well-being, which has a significant overlap (21–60%) with PANSS^115^. The present study is limited by the unavailability of a validated symptom severity measure for psychotic cohorts, such as PANSS, in view of the heterogeneity of the patient cohort.

In conclusion, the present study describes a whole blood mtDNA copy number reduction with increasing psychosis severity, potentially driven by the use of antipsychotic drugs in those treated with clozapine and risperidone. The drug-dosage dependent reduction by clozapine and risperidone was present in both whole blood and human neurons after *in vitro* exposure. Our novel findings support earlier studies that reported mtDNA copy number reduction in blood from SZ and BD-I patients. However, before whole blood mtDNA copy number can be evaluated as a possible biomarker of psychosis or its progression, e.g. reflect psychosis-intrinsic mitochondrial changes, further research is required to estimate the relative contribution of other antipsychotic drugs and comorbidities to the whole blood mtDNA copy number.

## Electronic supplementary material

Supplementary Table S1 and Figures S1-S4

## Data Availability

The authors report no biomedical financial interests, non-financial interests or other competing interests. The authors adhere to the data availability policy of *Scientific Reports*.
