# Publisher Correction: Mitochondrial DNA copy number is associated with psychosis severity and anti-psychotic treatment

**DOI:** 10.1038/s41598-019-53159-5

**Published:** 2019-11-05

**Authors:** Parvin Kumar, Paschalis Efstathopoulos, Vincent Millischer, Eric Olsson, Ya Bin Wei, Oliver Brüstle, Martin Schalling, J. Carlos Villaescusa, Urban Ösby, Catharina Lavebratt

**Affiliations:** 1 Department of Molecular Medicine and Surgery, Karolinska Institutet, Stockholm, Sweden; 2 Center for Molecular Medicine, Karolinska University Hospital, Stockholm, Sweden; 3 Department of Adult Psychiatry, PRIMA Child and Adult Psychiatry AB, Stockholm, Sweden; 4 Department of Neurobiology, Care Sciences and Society, Karolinska Institutet, Stockholm, Sweden; 5 Department of Psychiatry, University of California San Diego, California, USA; 6 Institute of Reconstructive Neurobiology, University of Bonn Medical Faculty, Bonn, Germany

Correction to: *Scientific Reports* 10.1038/s41598-018-31122-0, published online 24 August 2018

In the original version of this Article, the author Ya Bin Wei was incorrectly indexed. This error has now been corrected.

