# Supplementary material for: Mitochondrial DNA copy number is associated with psychosis severity and anti-psychotic treatment
Source: Sci Rep. 2018 Aug 24;8:12743. doi: 10.1038/s41598-018-31122-0 (PMC6109159; doi:10.1038/s41598-018-31122-0)
Supplement: Supplementary file 1 — Supplementary Table S1 and Figures S1-S4 [file 41598_2018_31122_MOESM1_ESM.pdf]

Mitochondrial DNA copy number is associated with psychosis severity and anti-psychotic treatment.

Parvin Kumar[1,2,*], Paschalis Esthathopoulos[1,2], Vincent Millischer[1,2], Eric Olsson[3,4], Ya Bin Wei[1,2,5], Oliver Brüstle[6], Martin Schalling[1,2], J. Carlos Villaescusa[1,2], Urban Ösby[2,3,4], Catharina Lavebratt[1,2,*]

[1]Department of Molecular Medicine and Surgery, Karolinska Institutet, Stockholm, Sweden

[2]Center for Molecular Medicine, Karolinska University Hospital, Stockholm, Sweden

[3]Department of Adult Psychiatry, PRIMA Child and Adult Psychiatry AB, Stockholm, Sweden

[4]Department of Neurobiology, Care Sciences and Society, Karolinska Institutet, Stockholm, Sweden.

[5]Department of Psychiatry, University of California San Diego, California, USA

[6]Institute of Reconstructive Neurobiology, University of Bonn Medical Faculty, Bonn, Germany

Word count of the abstract: 180 **manuscript body**: 3746 **references**: 2934

The number of figures, tables and supplemental information:

3 Tables

1 figure

4 Supplementary figures and 1 Supplementary table

[*] **Correspondence**:

Catharina Lavebratt, Karolinska University Hospital L8:00, SE-171 76 Stockholm, Sweden.

E-mail: Catharina.lavebratt@ki.se; Tel: +46-8-51775541; Fax: +46-8-51773909

Parvin Kumar, Karolinska University Hospital L8:00, SE-171 76 Stockholm, Sweden. E-mail: parvin.kumar@ki.se; Tel: +46-8-51776524; Fax: +46-8-51773909

Supplementary Figures

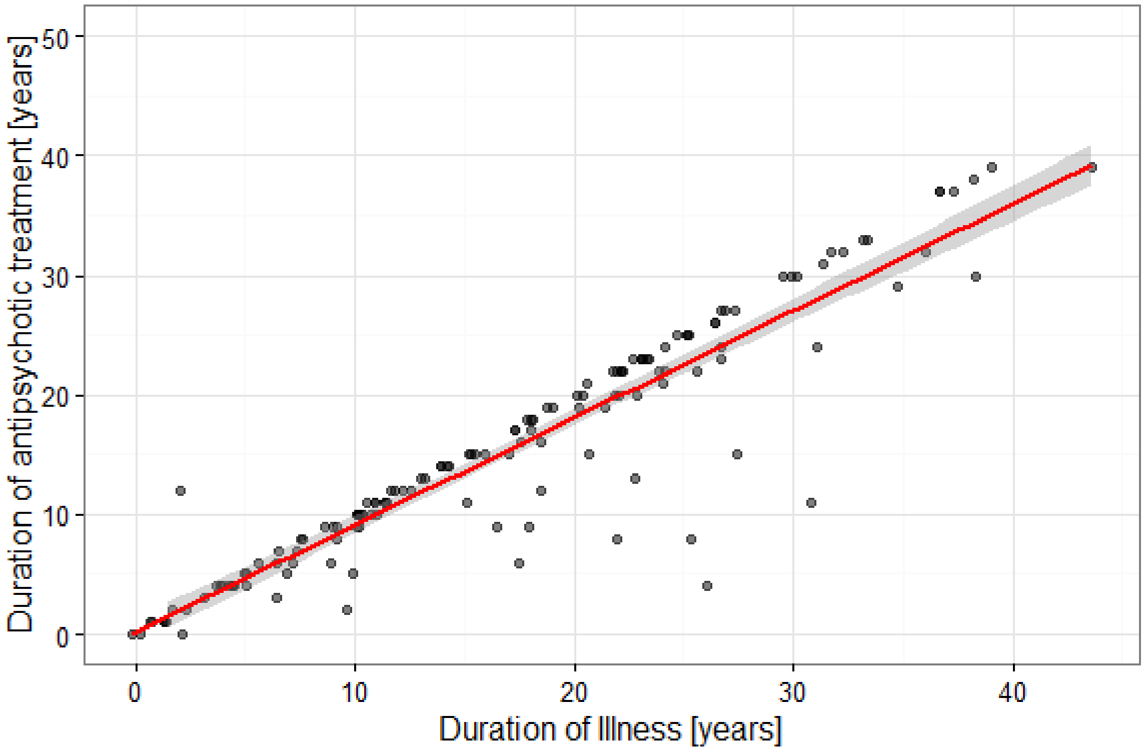

Figure S1A: Duration of antipsychotic treatment positively correlated with duration of illness within the cohort of patients on clozapine and risperidone (Spearman's $\rho = 0.915$, $p < 0.001$, $n = 145$).

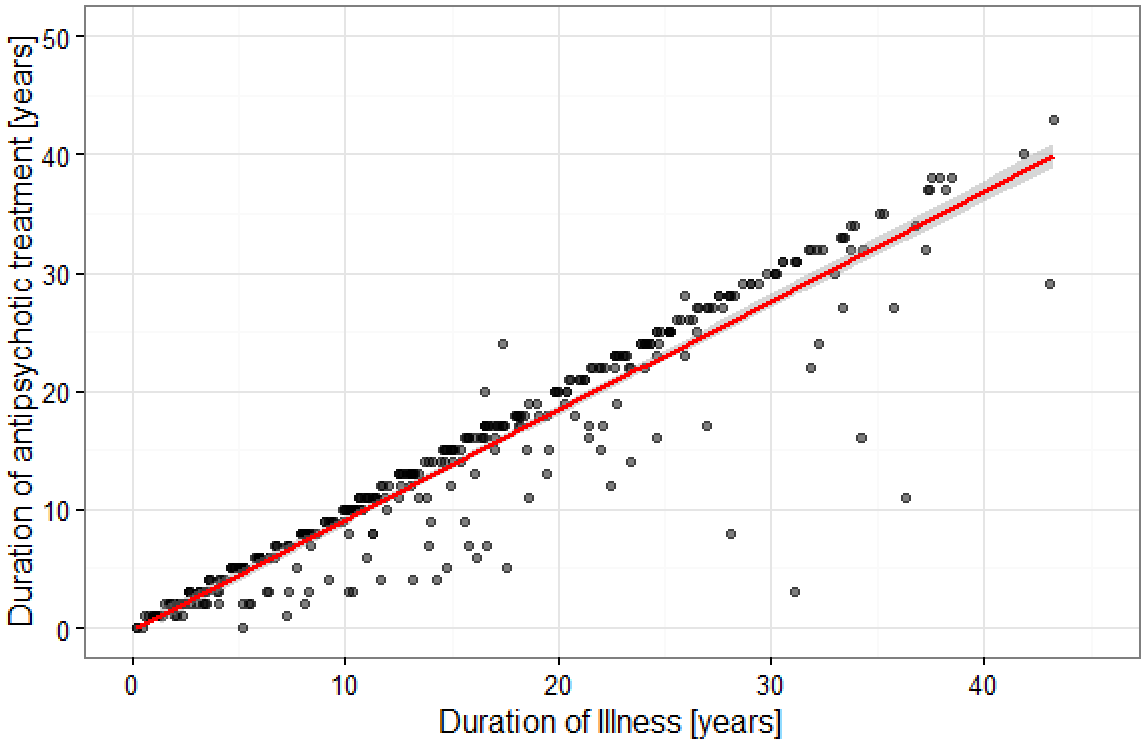

Figure S1B: Duration of antipsychotic treatment positively correlated with duration of illness within the cohort of patients not on clozapine and risperidone (Spearman's $\rho = 0.944$, $p < 0.001$, $n = 359$).

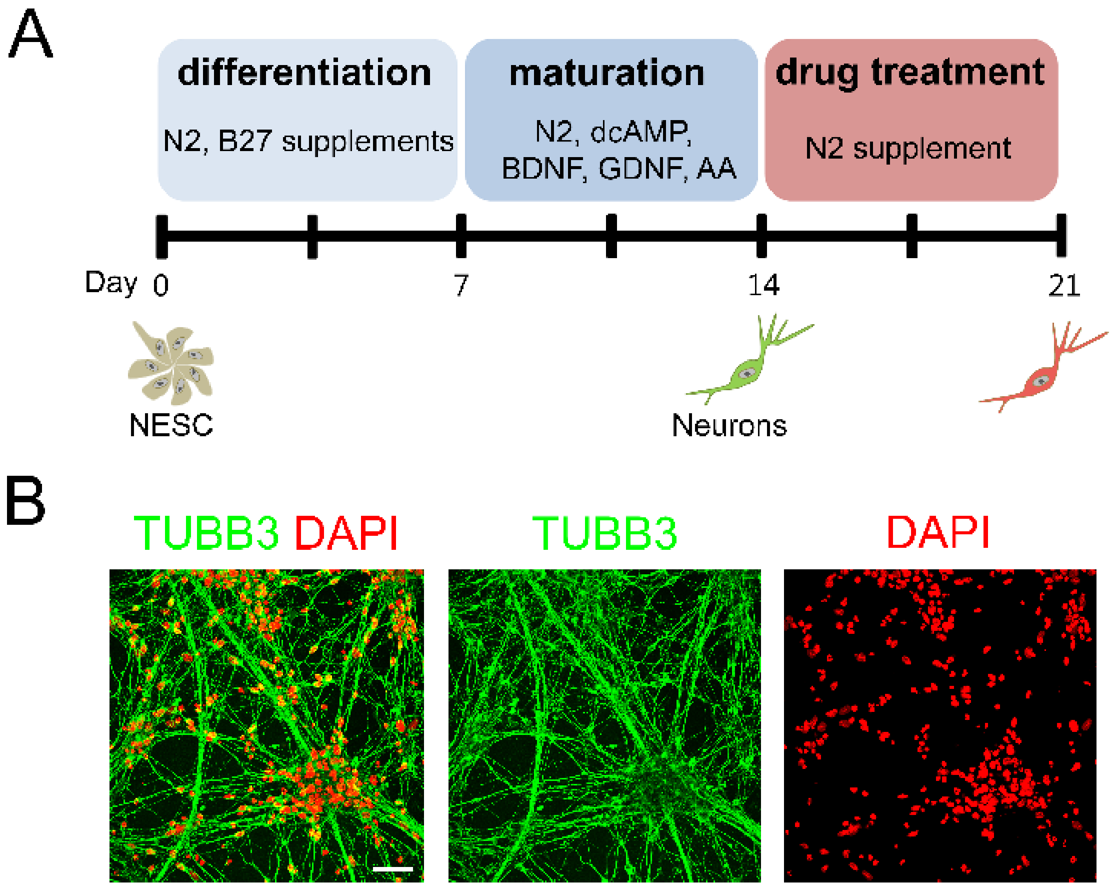

Figure S2A and S2B

In vitro effects of anti-psychotics on mitochondrial DNA copy number in neurons derived from human neuroepithelial stem cells (NESCs).

(A) NESCs were differentiated for 7 days with N2 and B27. They were maturated with N2, dcAMP, brain-derived neurotrophic factor (BDNF), glial cell-derived neurotrophic factor (GDNF) and ascorbic acid (AA). (B) Mature neurons' morphology was characterized through stains for neuronal marker TUBB3, white scale bar: 50 µm.

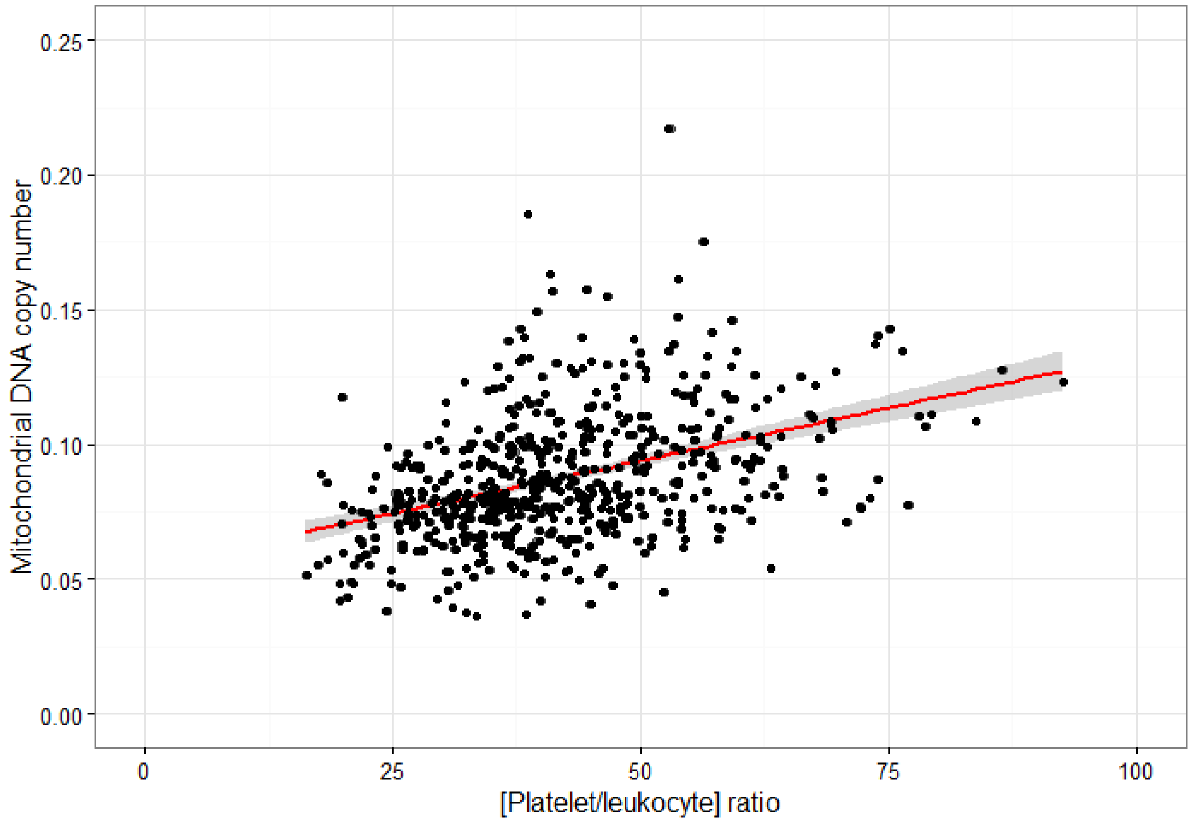

Figure S3: Whole blood mtDNA copy number was associated with the platelet to leukocyte count ratio (ρ = 0.422, p=2.2e-16). Therefore, mtDNA copy number was regressed on [platelet/leukocyte] ratio to obtain normally distributed unstandardized residuals. The residual variable designated $mtDNAcn_{res}$ represents the variance of whole blood mtDNA copy number that is unbiased by platelet to leukocyte count.

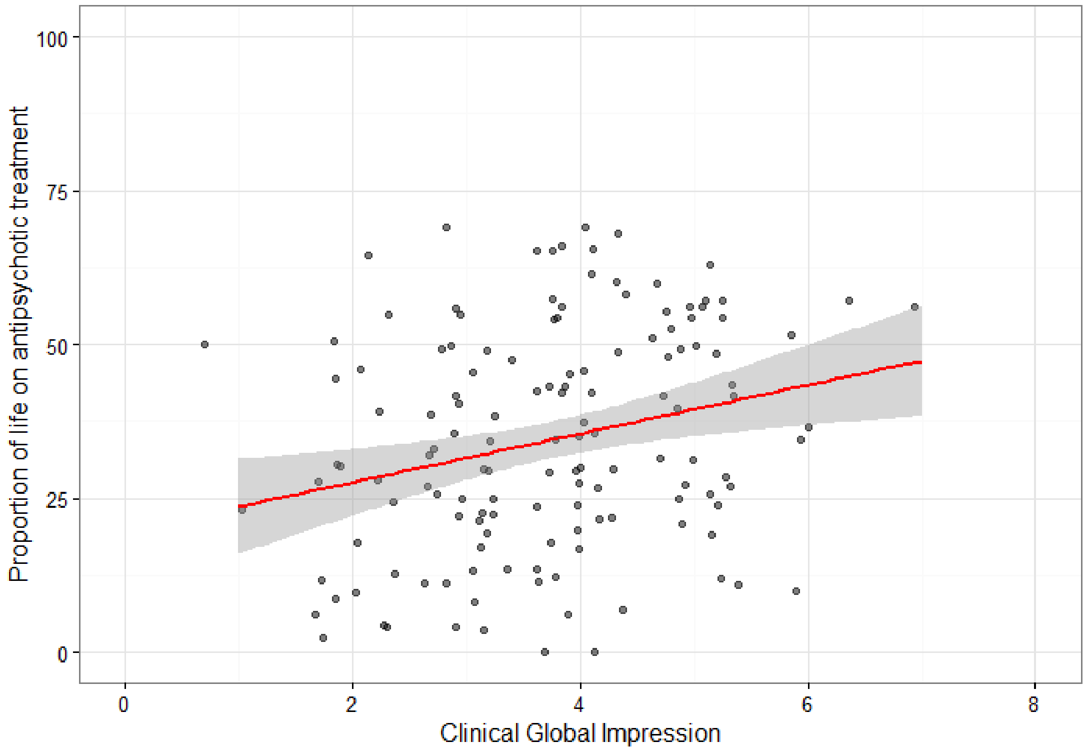

Figure S4A: Proportion of life on antipsychotic treatment positively correlated with Clinical Global Impression CGI (score) within the group of patients on treatment with clozapine and risperidone (Spearman’s $\rho = 0.258$ $p = 0.002$, $n = 140$ and $\beta = 0.248$).

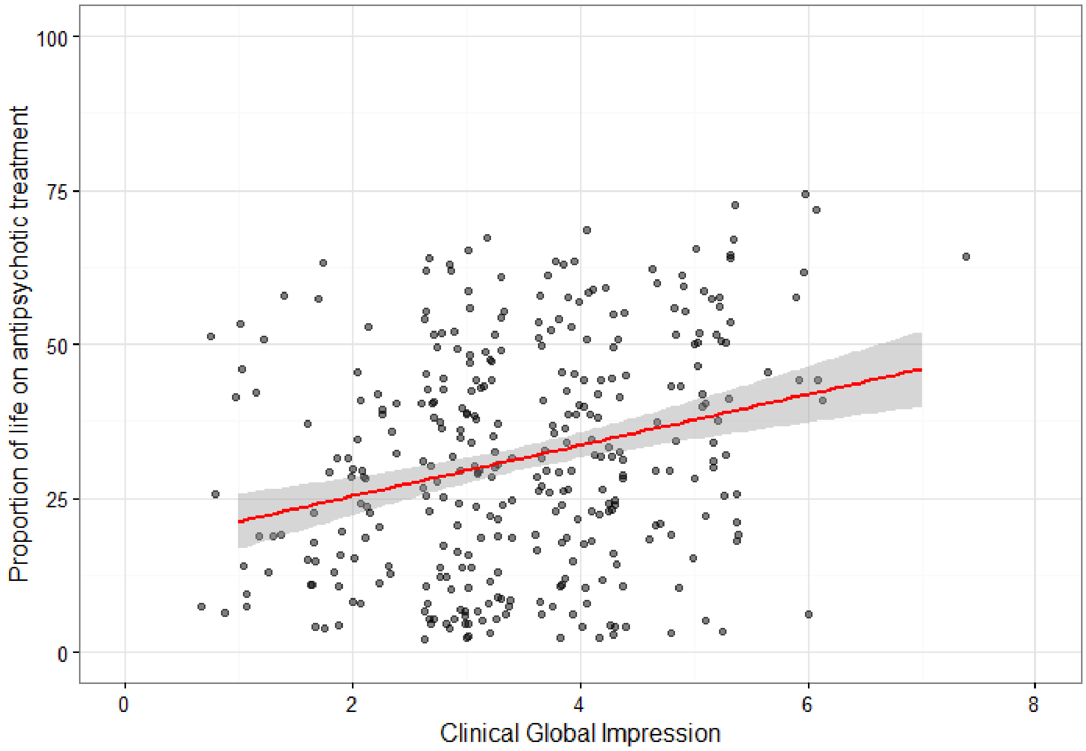

Figure S4B: Proportion of life on antipsychotic treatment positively correlated with Clinical Global Impression CGI (score) within the group of patients not on clozapine and risperidone treatment (Spearman's $\rho = 0.234$, $p < 0.001$, $n = 349$ and $\beta = 0.251$)

Table S1: Final models of clinical parameters associated with whole blood mitochondrial DNA copy number adjusted for platelet to leukocyte count. Analyses restricted to patients with Schizophrenia diagnosis, N=306.

**Variable**	**B**	**SE**	**$\beta_{standardized}$**	**t**	**p**
**Model 1 : Clinical profile (adjusted R^2 = 0.031, ANOVA: F= 5.65 , n =288)**					
(Constant)	0.117	0.010		12.2	<0.001
Age	<0.001	<0.001	-0.120	-2.071	0.039
CGI	-0.004	0.001	-0.145	-2.495	0.013
**Model 2 : Treatment (adjusted R^2 = 0.052, ANOVA: F = 5.65, n = 279)**					
(Constant)	0.114	0.009		13.1	<0.001
Age	-0.001	<0.001	0.170	-2.87	0.004
Clozapine	-0.013	0.005	-0.172	-2.89	0.004
Risperidone	-0.010	0.004	-0.139	-2.37	0.019
**Model 3 Group A : Treatment vs psychosis severity (adjusted R^2 = 0.040 , ANOVA: F = 5.02, n = 96)**					
(Constant)	0.088	0.055		19.1	<0.001
Proportion of life on treatment	-0.001	<0.001	-0.224	-2.24	0.027
**Model 3 Group B : Treatment vs psychosis severity (adjusted R^2 = 0.018 , ANOVA: F = 4.39, n = 190)**					
(Constant)	0.106	0.008		13.9	<0.001
CGI	0.004	0.002	-0.151	-2.10	0.037
**Model 4 Group A : Metabolic profile (adjusted R^2 = 0.049, ANOVA: F = 5.37 , n = 85)**					
Proportion of life on treatment	-0.001	<0.001	-0.245	-2.32	0.023
**Model 4 Group B : Metabolic profile (n = 204)**					
No significant predictors					

CGI: Clinical Global Impression-Severity

Group A: patients receiving clozapine or risperidone

Group B: patients not receiving clozapine or risperidone
